# Human in vitro models for understanding mechanisms of autism spectrum disorder

**DOI:** 10.1186/s13229-020-00332-7

**Published:** 2020-04-16

**Authors:** Aaron Gordon, Daniel H. Geschwind

**Affiliations:** 1 Department of Neurology, David Geffen School of Medicine, University of California Los Angeles, Los Angeles, CA USA; 2 Program in Neurobehavioral Genetics, Semel Institute, David Geffen School of Medicine, University of California, Los Angeles, CA USA; 3 Center for Autism Research and Treatment, Semel Institute, David Geffen School of Medicine, University of California, Los Angeles, CA USA; 4 Department of Human Genetics, David Geffen School of Medicine, University of California, Los Angeles, CA USA

## Abstract

Early brain development is a critical epoch for the development of autism spectrum disorder (ASD). In vivo animal models have, until recently, been the principal tool used to study early brain development and the changes occurring in neurodevelopmental disorders such as ASD. In vitro models of brain development represent a significant advance in the field. Here, we review the main methods available to study human brain development in vitro and the applications of these models for studying ASD and other psychiatric disorders. We discuss the main findings from stem cell models to date focusing on cell cycle and proliferation, cell death, cell differentiation and maturation, and neuronal signaling and synaptic stimuli. To be able to generalize the results from these studies, we propose a framework of experimental design and power considerations for using in vitro models to study ASD. These include both technical issues such as reproducibility and power analysis and conceptual issues such as the brain region and cell types being modeled.

## Early development as a critical period for ASD susceptibility

Several emerging lines of evidence have established that disruption of prenatal brain development is a major risk pathway for development of autism spectrum disorder (ASD) [1–3]. Many of the genes found to be associated with ASD are highly co-expressed in both neural progenitors and newborn neurons and peak in expression during prenatal brain development [1–4]. Moreover, work integrating genome wide association study (GWAS) results [1] with gene regulatory interactions, including expression quantitative trait loci (eQTL) [5] and 3D chromatin structure, demonstrates enrichment of ASD risk alleles in human-specific gene enhancers active in fetal brain and, in particular, in neural progenitors [6].

At the neuropathological level, studies have identified abnormalities in cerebral cortex in individuals with ASD, including smaller neurons, a higher abundance of neurons, ectopic cells, and dendritic abnormalities, which are likely to be caused by abnormalities in cortical development [7]. Neuroimaging studies found changes in cortical surface area in ASD as early as 6 months postnatally, likely due to prenatal abnormalities in cortical development [8]. Another line of evidence comes from studies of environmental exposures associated with ASD. These include studies associating prenatal exposure to valproate [9], as well as to maternal bacterial [10] and viral infections during pregnancy (i.e., cytomegalovirus (CMV)) [11]. These diverse lines of evidence implicate early cortical development as one major convergent period of risk in the development of ASD. Even more remarkably, late onset disorders such as schizophrenia and bipolar disorder, as well as non-specific risk for neuropsychiatric disorders, have also been linked to fetal brain development—emphasizing the importance of development in susceptibility for psychiatric disorders more broadly [12–16].

## Animal models

In vivo animal models are a major avenue of research for studying early brain development and how it is altered in ASD [17–21]. These models have many advantages as they can be used to study the entire process of brain development including age-dependent pathophysiology [21]. They allow for manipulation of specific genes on a homogeneous genetic background thus offering a way to study the effects of specific genes on the transcriptome, cell and circuit function, brain network activity, and behavior [17–21]. However, mouse models do not capture primate-specific or human-specific mechanisms active during early brain development or human complex genetic risk [22]. These human-specific mechanisms include many regulatory events, such as enhancer function and enhancer-promoter interactions, which govern gene expression in human neurogenesis and neurons [6, 13, 23–27].

Primate models are being developed to address some of these issues with mouse and other rodent models [28–30]. However, these primate models are expensive to develop and maintain, have a long reproductive cycle, and require careful ethical consideration [29]. Additionally, like mouse models, these models cannot yet capture genetic background effects or the polygenic contribution to ASD [31].

## In vitro options for studying human brain development

In vitro models allow researchers to model typical early human brain development, as well as changes occurring in ASD and other neurodevelopmental disorders (NDDs) [32]. The advantages and disadvantages of the most widely used methods have been extensively reviewed [32–39] and are briefly summarized in Table 2. We provide an overview of the various major techniques below, which can broadly be categorized into three major groups based on the source of the cells used.

The first method utilizes primary human neural precursor cells (phNPCs) extracted from fetal postmortem cortex. These phNPCs are aggregated into neurospheres which can be cultured for extended periods of time [40]. These neurospheres are further differentiated into neurons and glia using combinations of growth factors [40, 41]. The resulting neurons closely model in vivo fetal cortical development up to mid-gestation (19–24 post conception weeks) [41]. The expression of a group (module) of genes harboring de novo loss of function mutations in ASD and related to chromatin remodeling in vivo was well preserved in phNPCs [41]. These results are consistent with data indicating that chromatin structure in these neurons, as queried by ATAC-seq, highly overlaps with in vivo patterns [12].

The second method, termed trans-differentiation, directly induces neurons (iNs) from non-neuronal cells by using combinations of induction factors which activate a neuronal transcription cascade [42, 43]. This method, which often uses combinations of transcription factors, can quickly generate many types of iNs from somatic cells and results in a mature post-mitotic population of iNs without going through a neural progenitor (NPC) stage [42, 43]. These iNs retain many of the epigenetic marks of the source tissue [44, 45] which can capture the epigenetic signature of aging. This can be advantageous, for example, when studying neurodegenerative diseases [46, 47]. Given this method’s speed and reliability of generating post mitotic maturing neurons, direct induction approaches can be advantageous, especially in the context of high throughput screens for which speed and reliability are paramount [42, 43, 48–55]. However, this method does not allow for complete and faithful modeling of fetal neuronal development, which depends on the correct sequence of developmental steps and epigenetic signature [44, 45, 56, 57].

The third method relies on embryonic (ESC) or induced pluripotent stem cells (iPSC) which are differentiated into heterogeneous cultures and can recapitulate different in vivo developmental stages [58]. One advantage of using iPSCs over ESC is that they can be generated from cells collected directly from individuals with ASD and can thus be used to capture both the genetic background, as it may influence major effect mutations, as well as idiopathic forms of ASD [32]. Another advantage is that the findings from iPSC derived from individuals with ASD can be integrated with available medical records, imaging results, and family pedigree which could supply the study with valuable phenotypic data. One example of this integrated head size as a phenotype to study changes occurring in individuals with ASD and macrocephaly [59, 60]. These advantages are often also true for iNs derived from patients [39]; however, unlike iNs, iPSCs can recapitulate different in vivo developmental stages and have a methylation profile which resembles that of ESC [61–65]. It is important to note that iPSC do retain a small fraction of methylation markers from the donor, which can differ between different iterations of reprogramming and can depend on the source of the reprogrammed cells used [62, 63, 65]. Additionally, iPSCs tend to have lower genetic stability, sometimes leading to multiple unintended copy number variants (CNV) and single nucleotide variants (SNV), which necessitates whole genome sequencing to validate each line [66].

Both ES and iPSC can be differentiated into 2-dimensional (2D) and 3-dimensional (3D) neuronal cultures. 2D cultures can be generated by adding growth factors [67, 68] or small molecules [69, 70] to the medium to generate NPC, which can then be further differentiated in neurons [39]. Direct differentiation into neurons which does not go through a NPC stage, as mentioned above, can also be achieved by overexpressing growth factors (e.g., NGN2, or Ascl1/Dlx2) [71, 72]. This can result in a more homogenous cell population and is highly scalable and reproducible [71, 72]. However, these mono-layer cultures do not fully capture in vivo brain development, as they lack the dense cellular environment of the brain which includes many synaptic and glial junctions [32, 39, 73]. Additionally, the direct to neuron methods may miss critical steps in the developmental trajectory of neurons where genetic risk may be acting, making them less suited to study neurodevelopment [39, 71].

3D cultures, also referred to as organoids, which capture more of the architecture (e.g., cortical layering) and cellular environment of in vivo brain development, can be organized by level of directed differentiation going from less directed to highly directed differentiation [32, 33, 38, 74–76]. While all differentiations are initially grown in neural induction media, in the less directed differentiations, the cells are not directed to differentiate into a specific brain region using additional factors [75, 77–80]. These differentiation methods lead to cultures with a variety of brain regions which can be used to study inter- and intra-regional connections [75, 81, 82]. However, these methods require careful assessment, particularly when studying disease, as regional heterogeneity can make these cultures extremely variable, making it difficult to compare between different cultures, even those that are presumed replicates from the same individual [81]. In contrast, the more directed differentiation protocols use specific combinations of morphogens, signaling molecules and growth factors to guide the cultures to differentiate into a specific brain region (often dorsal forebrain). To promote neural induction, many of these protocols initially add different combinations of growth factors (i.e., EGF, NT3, BDNF, and GDNF) [74, 78, 79, 83, 84]. This results in more reproducible cultures compared to the less directed differentiation, as seen by lower variability and more consistent cell types and cell proportions [74, 83–86]. More recently, multiple groups have described fusing the more directed organoids from different brain regions together. These combined cultures, termed assembloids, model the development of complex interconnected regions thus more faithfully recapitulating in vivo development and function [85, 87, 88]. For example, the fusing of dorsal and ventral forebrain cultures has been shown to reliably integrate interneurons into the dorsal forebrain [85, 87, 88].

Work in 3D in vitro models of brain development is in its early stages and more work is needed to improve their ability to faithfully and reproducibly recapitulate in vivo development. A recent study noted that these cultures can show increased levels of cell stress as well as reduced cell subtype specification compared to in vivo [78]. A noteworthy disadvantage of these 3D cultures compared to 2D cultures expressing NGN2 [71] and iNs [42, 43] is that while cells in these 2D methods take roughly 2 weeks (14 days) to differentiate into neurons, 3D cultures typically take 2–4 months (60–120 days) to reach differentiation levels similar to mid-gestation [74–76, 78, 83]. This makes the 3D cultures less scalable and therefore less suited for large scale screens [32, 33]. These longer differentiations, however, can also be viewed as an advantage, as they can lead to more mature cellular and transcriptomic phenotypes in both neurons and glia [77, 84, 89].

## Applications

### Studying neurodevelopmental and psychiatric disorders

In vitro models can be used to study the effects of both common and rare genetic variation on early human brain development at a cellular and molecular level, in both typical development and neurodevelopmental and psychiatric disorders. Since most genetic variation resides in non-coding regions, which are highly diverged between human and rodents [90], it is necessary to use primate or human models to understand the role of most regulatory variation [5], especially for ASD risk genes that are regulated by human evolved elements [6]. Moreover, studying the role of common genetic background on neurodevelopment in ASD and other psychiatric disorders is currently only feasible by studying patient-derived cellular models.

Using these models, one can compare differentiated cultures from individuals with ASD and other psychiatric disorders, either from those without a clearly defined genetic etiology or from those with genetically defined forms. This approach accounts for the genetic background and, in the case of genetically defined forms, also integrates the effects of the mutation with the genetic background, giving results that can reflect the complex genetic architecture of these disorders. A complementary approach to study genetically defined forms is to use isogenic lines in which researchers either induce mutations in control lines or correct mutations in lines derived from individuals with ASD, e.g., using CRISPR/Cas9 methods [91]. This approach minimizes variation caused by genetic background and directly links the observed phenotype with the mutation [91]. Thus, it allows for direct inference of the role that ASD risk genes play in neurodevelopment.

In addition to understanding both common and rare genetic risk, iPSC-derived models can also be used to study the role of environmental factors on both typical and atypical brain development. These environmental factors can be extrinsic, such as organophosphates [92] and bisphenol-A [93], or in utero factors such cortisol levels and inflammatory factors [94, 95]. For example, exposing neuroepithelial-like stem cells to high levels of glucocorticoids for 48 h transiently increased intracellular reactive oxygen species concentration [96]. This exposure led to persistent inhibition of neural differentiation and increase in glial differentiation [96].

### Precision medicine

Stem cell models of brain development can also be used in the field of precision medicine [97]. Stem cell-derived neurons could serve as a potential diagnostic tool for enigmatic rare diseases. In cases where whole exome sequencing does not yield a diagnosis, transcriptomic analysis of relevant tissue has shown some promise [98–101]. In cases when the relevant tissue is inaccessible (which is the majority of cases in neurodevelopmental and psychiatric disorders), blood transcriptomics sequencing has been suggested as an alternative and was shown to be informative in 7.5–16.7% of cases [101, 102]. However, many cases still remain undiagnosed, and it is reasonable to believe that transcriptomics sequencing from cells mimicking the relevant tissue by using stem cell-derived cultures would further increase this rate of diagnosis.

Additionally, given the high heterogeneity and polygenicity of psychiatric disorders, these in vitro models could help identify intermediate processes leading to neuronal dysfunction [103]. Combined with genetic data, medical record data, and imaging results, this could lead to stratification of patient populations into more homogenous cohorts and to development of cohort-specific treatments [97].

### Drug discovery

Stem cell-based models can also be used to screen drugs for treatment of neurodevelopmental disorders including ASD [91, 104]. For example, one study screened 4421 unique compounds and identified 108 compounds that regulate neurite growth [105], a process which has been variably linked to some forms of ASD [106]. Another study screened a set of 50,000 compounds in neural stem cells to find activators of *FMR1*, a gene silenced in fragile X syndrome, which increases risk for ASD [107]. Similarly, a different study screened 202 compounds for their ability to restore *SHANK3* expression in *SHANK3* haplo-insufficient stem cell-derived neurons [108]. Two compounds, lithium and valproic acid (VPA), were found to restore *SHANK3* expression and increase network connectivity in these neurons [108].

Additionally, stem cell-derived neurons from individuals with psychiatric disorders can be used for drug discovery and for tailoring drug regimens to specific individuals or subgroups. For example, reversal of hyperexcitability in iPSC-derived neurons from individuals with bipolar disorder was a good predictor for the responsiveness of these individuals to lithium therapeutics [109].

### Evolution of the human brain

One other interesting emerging application is to study the evolution of the human brain by comparing cultures derived from human to other non-human primates which share many of the transcriptional programs determining cell type in the developing cerebellar cortex [82, 83, 110, 111]. One study, using 2D and 3D stem cell-derived cultures, found that differences in neuronal cell numbers among rodents, non-human primates, and humans could be partially explained by the differences in the presence and length of a developmental stage of cerebral cortex progenitor expansion that was significantly increased in humans [111]. Supporting this finding, two studies found that cellular maturation took longer in humans organoids compared to chimpanzee and bonobo organoids [82, 110]. Many upregulated genes and changes in DNA accessibility in these studies were identified as being specific to the developing human brain [82]. Additional support to the extended maturation of human cells comes from co-expression network analysis which identified human-specific transcriptional changes in groups of genes related to cell cycle and neuronal apoptosis [83].

### Main findings from stem cell models of ASD to date

Two kinds of genetic modeling have been performed using cells either from individuals whose genetic contributions are unknown or undefined, so called idiopathic [59, 60, 112–120], or from individuals harboring major effect mutations that are presumed causal or which have been engineered to carry these mutations. These mutations include ASD-associated CNVs such as 15q11q13 deletion (Angelman syndrome) [121] and duplication (Dup15q syndrome) [122], 22q11.2 deletion (DiGeorge syndrome) [123, 124], 16p11.2 deletion and duplication [125], and 15q13.3 deletion [126], as well as single-gene mutations including *SHANK3* [127–130], *CHD8* [131, 132], *NRXN1* [133–137], *NLGN4* [138], *EHMT1* (Kleefstra syndrome) [139], *PTCHD1-AS* [140], *UBE3A* (Angelman’s syndrome) [141], and *CACNA1C* (Timothy syndrome) [142] (summarized in Table 1). In this review, we will not discuss fragile X syndrome, Rett’s syndrome, and tuberous sclerosis-related autism as they have all been extensively reviewed previously [148–154].

**Table 1:** Summary of studies using in vitro model to study ASD

Mutation type	Study	Gene/Syndrome	Number of individuals (ASD/Control)	Isogenic?	Proliferation	Cell death	Neuronal differentiation	Electrophysiological properties
Idiopathic	Mariani et al. [59]	Idiopathic with macrocephaly	4/8	No	Decreased cell-cycle length in NPCs and early stages of neuronal differentiation	NA	Increased MAP2 in neurons More synaptic puncta Increased proportion of GABAergic neurons	Reduced peak sodium current
	Liu et al. [115]	Idiopathic (no seizures or ID)	3/3	No	NA	NA	No change in cell proportions No difference in the number of primary neurites	Reduced sEPSC frequency and half width while amplitude and rise time were not changed Decreased Na+ and fast K+ voltage-gated currents
	Marchetto et al. [60]	Idiopathic with macrocephaly	8/5	No	NPCs proliferated faster	NA	Reduced proportion of BRN2+ and NGN2+ cells Increased proportion of GABAergic cells Fewer excitatory glutamatergic synapses Reduced maturation	No difference was observed in the frequency of spontaneous action potentials Reduced number of synchronized bursts No increase in the number of spikes with maturation
	Russo et al. [116]	Idiopathic (without seizures)	3/3	No	No differences in proliferation	NA	No change in cell proportions Reduced synaptogenesis as an interaction between astrocytes and neurons	Decreased spontaneous spike rate
	DeRosa et al. [112]	Idiopathic	5/5	No	NA	NA	NA	Fewer spontaneous spikes Fewer spontaneous calcium transients
	Griesi-Oliveira et al. [113]	Idiopathic	13/8	No	NA	NA	NA	NA
	Schafer et al. [114]	Idiopathic	8/5	No	NA	NA	Acceleration differentiation of neurons More complex neurite branching patterns	NA
	Lewis et al. [143]	Idiopathic	1 multiplex family - 1 affected individual/1 intermediate phenotype relative/1 unaffected relative and 1 unaffected control	No	No changes in cell cycle	Increased apoptosis of both excitatory and inhibitory neurons	DEG were enriched for GO terms related to neuron differentiation	NA
	Moore et al. [118]	Idiopathic	3/3	No	Increased proliferation	NA	Decrease in the proportion of neurons Shorter neurites	NA
	Adhya et al. [117] preprint	Idiopathic/*NRXN1*/3p deletion syndrome	6/2/1/3 controls	No	Upregulation of genes associated with cell proliferation	Upregulation of genes associated with regulation of apoptosis	Delayed neuronal maturation Fewer excitatory and inhibitory NPCs but more GABAergic neurons	NA
	Wang et al. [119]	Idiopathic with macrocephaly (subset of [60])	3/3	No	Increased proliferation leading to an increase in double stranded breaks	No	Decreased cell migration Defects in polarity and adherence junctions	NA
	Griesi-Oliveira et al. [120]	Idiopathic	6/6	No	Upregulation of genes associated with cell proliferation in NPCs	NA	Upregulation of genes associated with synapse and neurotransmitter release Shorter neurites with fewer ramifications	NA
	Deshpande et al. [125]	16p11.2 deletion and duplication	3 deletion/3 duplication/4 control	No	No changes in proliferation	NA	16p11.2 deletion neurons have increased total dendrite length and more extensive dendritic arbors compared with controls The 16p11 duplication neurons exhibit the opposite phenotype with significantly reduced total dendrite length Lower density of synaptic puncta, in both 16p11.2 deletion and 16p11.2 duplication	Reduced excitability in 16p11.2 deletion No difference in the kinetics or frequency of the mEPSCs in 16p11.2 deletion and 16p11 duplication neurons
CNV	Lin et al. [144]	22q11.2 deletion (DiGeorge syndrome)	8/7	No	Downregulated DEGs were enriched for cell cycle GO terms Decreased proliferation	Upregulated DEGs were enriched for apoptosis	NA	NA
	Toyoshima et al. [124]	22q11.2 deletion (DiGeorge syndrome)	2/3	No	Smaller neurospheres	NA	The fraction of neurons was reduced while the fraction of astrocytes was increased Shorter neurites	
	Fink et al. [122] preprint	Dup15q syndrome (15q11-q13 duplication) Angelman syndrome (15q11-q13/UBE3A maternal deletion) 15q11-q13 paternal duplication	4 Dup15q/3 Angelman syndrome/1 paternal duplication/6 controls	No	NA	No change in cell death in Dup15q	No differences in dendritic complexity in Dup15q Decrease dendritic complexity in Angelman syndrome	Delayed maturation of action potential Increased frequency and amplitude of synaptic events Increased frequency of spontaneous firing of action potentials
	Fink et al. [121]	Angelman syndrome (15q11-q13/*UBE3A* maternal deletion)	3/4	Yes	NA	No change in cell death	No changes in cell proportions	Impaired maturation of resting membrane potential Decreased spontaneous excitatory synaptic activity
	Gillentine et al. [126]	*CNRNA7*(15q13.3 deletion) deletion and duplication	3 duplication/3 deletion/3 control	No	NA	NA	NA	Decreased α7 nAChR-associated calcium flux in both deletions and duplications
	Deneault et al. [145]	16p11.2 deletion, Nrxn1, *DLGAP*, *CNTN5*, *AGBL4*, *GLI*, *CAPRIN*, *VIP*, *ANOS1*, *EHMT2*, THRA, *SET*	53 lines from 26 individuals 15 ASD/11 control (1 individual from each, 2 Mz from SET)	Yes	NA	NA	NA	Increased neuronal activity in glutamatergic neurons with CNTN5 or EHMT2 mutations
Single gene	Pasca et al. [142]	*CACNA1C* (Timothy syndrome)	2/3	No	NA	NA	Decreased fraction of neurons expressing lower-layer markers and an increased fraction of neurons expressing upper-layer markers. More neurons expressed tyrosine hydroxylase (TH) which was not caused by shift in cell fate	No differences in the action potential threshold or amplitude, resting membrane potential, input resistance or capacitance Wider action potentials Increase in the sustained Ca2+ rise after depolarization
	Wang et al. [131]	*CHD8*	1 individual 2 control lines/4 heterozygous lines (Crispr)	Yes	NA	NA	DEG were enriched for neurogenesis, neuronal differentiation and forebrain development	NA
	Sugathan et al. [132]	*CHD8* (knockdown)	1 individual	Yes	NA	NA	Downregulated DEG were enriched for synapse formation and neuron differentiation	NA
	Frega et al. [139]	*EHMT1* (Kleefstra syndrome)	3/3	Yes	NA	NA	No difference in dendritic morphology or synaptic density	No differences in AMPA-related mEPSCs Fewer, less regular, and longer network bursts which were caused by NMDAR activity
	Marro et al. [138]	*NLGN4*	1 ES line/1 knockout line/1 mutant line	Yes	NA	NA	Increased number of excitatory synapses in mutant line	Increased frequency and amplitude of mEPSCs in mutant line
	Zeng et al. [134]	*NRXN1*	1 ES line/1 iPSC line	Yes	NA	NA	Downregulation of astrocyte differentiation DEG were enriched for neuron differentiation-related GO terms	NA
	Pak et al. [133]	*NRXN1*	2 induced mutations in 1 line	Yes	NA	NA	No changes in differentiation No changes in synaptic density No change in the number if readily releasable pool of vesicles	No change in intrinsic membrane properties Decreased frequency of spontaneous mEPSCs Impaired evoked neurotransmitter release
	Lam et al. [135]	*NRXN1*	1/4	No	Slower proliferation	NA	Decreased excitatory neuronal maturation Higher proportions of astroglia	Action potentials had lower amplitude and lower rise time Lower calcium concentration in response to depolarization
	Avazzadeh et al. [136]	*NRXN1*	3/5	No	NA	NA	No difference in cell proportions	Increased frequency, duration, and amplitude of calcium transients associated with action potentials
	Flaherty et al. [137]	*NRXN1*	4/4	Yes	NA	NA	Decreased proportion of mature neurons Decreased neurite number and length	Decrease in the number of spontaneous action potentials (using two maturation methods)
	Ross et al. [140]	*PTCHD1-AS*	2/2	Yes	No changes in proliferation	NA	Increased number of synapses in one of the subjects and decreased dendrite complexity in the other	No changes in membrane potential Decreased frequency of mEPSCs Decreased NMDA-evoked current amplitude
	Zaslavsky et al. [146]	*SHANK2*	2/4	Yes	Downregulation of cell cycle genes	NA	Increased synapse numbers, dendrite length, and neuron complexity Increased number of functional excitatory connections	Increased sEPSC frequency
	Yi et al. [127]	*SHANK3*	1 line	Yes	NA	NA	Decrease length and branching of neurites No change in the density or size of synapses	Increased input resistance with no change in capacitance Decreased evoked excitatory postsynaptic currents Decreased amplitude of spontaneous miniature EPSCs Hyperexcitability caused by impaired Ih currents
	Kathuria et al. [128]	*SHANK3*	2/3 1 ES line	Yes	NA	NA	Smaller cell soma and more primary neurites in olfactory placodal neurons but not in cortical neurons Shorter neurites in cortical neurons	NA
	Gouder et al. [129]	*SHANK3*	4/3	No	NA	NA	Reduced dendritic spine densities and spine volume	NA
	Huang et al. [130]	*SHANK3*	2 lines	Yes	NA	NA	Reduced neuronal soma size, neurite length, and neurite branch number	Reduced frequency of sEPSC
	Sun et al. [141]	*UBE3A* (Angelman’s syndrome)	1 ES cell line	Yes	NA	NA	No changes in neuron morphology	Increased firing frequency of action potentials Increased synchronization
	Deneault et al. [147]	Many genes (isogenic) *AFF2*/*FMR2*, *ANOS1*, ASTN2, *ATRX*, *CACNA1C*, *CHD8*, *DLGAP2*, *KCNQ2*, *SCN2A*, *TENM1*	1 control individual/1 line	Yes	NA	NA	NA	Reduced sEPSCs and in 5 out of 10 mutations often (4 out of 5 mutations) accompanied by reduced burst frequency

*Abbreviations*: *DEG* differential expressed genes, *EPSC* excitatory postsynaptic current, *sEPSC* spontaneous excitatory postsynaptic current, *mEPSC* miniature excitatory postsynaptic current, *NPC* neuron precursor cell

The majority of these studies used cells from patients, with some also including isogenic controls [121, 127, 131, 134, 145, 146], while some studies exclusively used induced mutations comparing them with isogenic control lines [132, 133, 138, 141, 147] (Table 1). Many of the studies, from both idiopathic and genetically defined forms of ASD, found effects in one (or more) of four general categories of cellular biological processes: (1) cell cycle and proliferation, (2) cell death (specifically apoptosis), (3) cell differentiation and maturation, and (4) neuronal signaling and synaptic stimuli (Table 1). We have therefore organized the results according to these categories.

### Cell cycle and proliferation

Several studies found changes in cell cycle in cells derived from both individuals with idiopathic or genetically defined forms of ASD. Neuronal cultures from individuals with idiopathic ASD and macrocephaly displayed accelerated cell cycle progression, accompanied by upregulation of genes involved in cell proliferation in several independent studies, making this one of the few findings to have been replicated [59, 60, 117, 119]. Two studies also found that neurons derived from individuals with ASD but without macrocephaly also proliferated faster [118, 120]. Conversely, genetically defined forms of ASD, mutation in *NRXN1*, and 22q11.2 deletion showed evidence of a decreased proliferation rate [124, 135, 144]. However, it is important to note that not all studies that examined cell cycle found changes in ASD [116, 125, 140, 143].

The acceleration in cell cycle in idiopathic ASD supports a finding from toddlers with ASD in which cell cycle gene networks were positively correlated with brain volume [155]. This acceleration could explain the differences in neuronal number and brain growth across the life span of individuals with ASD [156], as well as the high prevalence of macrocephaly in individuals with ASD [157]. However, an important caveat to this finding is that these changes in cell cycle could be an artifact stemming from confounders within culturing conditions that are propagated due to small sample size. To address this, larger samples are needed with robust measures for the reproducibility of the culturing system.

### Cell death

Studies in 22q11.2 deletion [144], as well as idiopathic forms of ASD [117, 143], found an increase in cell death—more specifically in apoptotic cell death—in mature neurons [117, 143, 144]. This increase in apoptotic cell death has also been described in vivo in a small sample of postmortem brains from children with idiopathic forms of ASD [158], a finding which has yet to be more broadly investigated.

### Neuronal differentiation and morphology

Studies on neurons derived from individuals with idiopathic ASD show conflicting results relating to neuronal differentiation. One study performed on individuals with macrocephaly found a general increase in the number of neural precursor cells (NPCs) [60]. This increase was driven by an expanded proportion of GABAergic inhibitory precursors, which unexpectedly led to a reduced number of GABAergic neurons [60]. Compared to GABAergic inhibitory precursors, the proportion of glutamatergic precursors was reduced in these cultures and was accompanied by a decrease in the number of excitatory synapses [60]. The increase in the total number of neurons was replicated in another study using 3D cultures, which also found accelerated development in differentiating excitatory neurons and more complex neurite branching patterns [114].

However, not all studies have found the same changes in cell proportions. One study, also based on individuals with macrocephaly, found an *increase* in GABAergic cell number accompanied by an increased number of GABAergic synapses [59]. This study did not find any changes in the number of excitatory glutamatergic neurons and synapses [59]. More recently, this increase in GABAergic neurons but not glutamatergic neurons, has been partially replicated from non-macrocephalic individuals with ASD, finding an increase in GABAergic cell markers, but no long-term changes in glutamatergic cell markers [117]. Yet, another study found a decrease in the total number of neurons in cultures from individuals with ASD without macrocephaly [118]. Contrary to the studies above which found some changes in cell proportions, a study performed using iPSCs from individuals with idiopathic ASD [116] found no change in cell proportions, but rather observed a reduction in glutamatergic synaptogenesis. This reduction was attenuated by the astrocytes in the culture as it was only seen when both neurons and astrocytes were derived from the individuals with ASD but not when the astrocytes were derived from healthy individuals [116].

These often conflicting results likely arise from many factors, ranging from etiological diversity, to small sample sizes, to differences in the culturing conditions. Unless one controls for the extraordinary etiological/genetic heterogeneity by studying known mutations, biological differences between a handful of different individuals with idiopathic ASD would likely swamp subtle differences in in vitro development, especially given the small effects sizes found in imaging studies [159]. The small sample sizes used in these studies (3–8 affected individuals per study) could also be a cause for these contradictory results. Small sample sizes have lower power to detect changes, tend to overestimate effect sizes, and can lead to low reproducibility [160]. Different culturing methods could also lead to very different results even when looking at the same individuals. This was demonstrated in one study, where the ASD phenotype of neurite complexity and length was completely dependent on the differentiation protocol [114]. When the neurons were generated via NPCs using extrinsic signals, an increase in neurites was observed, whereas when differentiating the cells directly into neurons by overexpressing *NGN2*, this phenotype could no longer be seen [114]. In another study that also highlighted the importance of culturing protocols, specifically the cell composition of these cultures, the source of the astrocytes co-cultured with neurons (control or ASD) had a large effect on the neuronal phenotype [116], demonstrating the importance of considering the extracellular environment and cell-cell communication in modeling development. To make the results from these studies more robust, one would ideally like to see larger, more well-powered studies and use of different culturing systems that best mirror in vivo development. Going beyond technical reproducibility, the contradictory findings in the literature emphasize that biological and genetic variability need to be better accounted for to be able to generalize the results. In summary, given the large heterogeneity and small effect sizes seen in these studies, combined with their relatively small sample sizes and variability in culturing methods, we find it difficult to generalize from any of the published findings based on studies of small numbers of patients with idiopathic ASD.

In contrast to the variable results in idiopathic ASD, findings from genetically defined forms of ASD are generally more coherent. This is consistent with the viewpoint that the phenotypic variability seen in the idiopathic forms of ASD is due, at least partially, to etiological diversity. Several genetic forms of ASD show a decrease in the number of neurons and synapses, including Timothy syndrome—in which there was a decrease in the fraction of neurons expressing lower layer markers [142] and 22q11.2 deletion, which showed a reduced number of neurons accompanied by an increase in the number of astrocytes [124]. Three studies on *NRXN1* mutations also found evidence for a decrease in neuronal maturation [134, 135, 137], a finding which was not replicated in a different study [136]. Similar results (downregulation of neuronal processes) were indirectly observed using transcriptomic analysis from neurons in which *CHD8* was either knocked down [132] or heterozygously deleted [131].

Neuronal morphology, and more specifically dendritic tree morphology, was also perturbed in many of the genetically defined forms of ASD. The size and complexity of the dendritic tree was decreased in neurons with *SHANK3* [127–130]. One study also showed a reduction in spine density [129], though this result was not replicated by a different group [127]. Similar decreases in dendritic tree complexity were also found in neurons derived from individuals with Angelman syndrome [122] and in one individual with a *PTCHD1-AS* mutation [140]. However, not all genetic forms of ASD followed this pattern of decreased complexity of the dendritic tree. Notably, the 16p11.2 locus shows a dosage effect on the size and complexity of the dendritic tree [125]. The dendritic length was decreased in 16p11.2 deletion and was increased in 16p11.2 duplication [125]. Additionally, in contrast to the findings with *SHANK3*, *SHANK2* loss of function mutations led to an increase in the number of synapses, as well as in the complexity of the dendritic tree [146]. Individuals with *NLGN4* [138] and one individual with a *PTCHD1-AS* mutation [140] also showed an increase in the number of synapses.

Interestingly, similar to the findings in some of these stem cell models, gene sets related to neurons and synaptic activity are downregulated in the postmortem cortex of individuals with ASD [120, 161–166] suggesting a possible point of convergence between some of the genetically defined and idiopathic forms of ASD.

### Neuronal signaling and synaptic function

Dysregulation in neuronal differentiation and synaptic and dendritic deficits may underlie the decreased spontaneous activity and decreased excitability found in many studies. These are often observed in neurons derived from individuals with idiopathic forms of ASD [112, 116, 167], as well as from individuals with genetically defined forms of ASD such as *SHANK3* [127, 130], 16p11.2 deletion and duplication [125], Angelman syndrome [121], Dup15q syndrome [122], *NRXN1* mutations [133, 135, 137], and PTCHD1-AS [140]. Decreases in spontaneous neuronal activity were also found in five out of ten genes associated with ASD when mutations were introduced into neurons derived from typically developing individuals (ATRX, AFF2, KCNQ2, SCN2A, and ASTN2; see Table 1 for the full list of genes tested) [147].

The evidence for decreased neuronal activity overlaps with findings from transcriptomic analysis of postmortem cortex from individuals without a clearly defined genetic etiology and individuals with Dup15q [161, 165, 166]. These analyses found downregulation of gene modules related to synaptic activity and neuronal firing [161, 165, 166]. Combining the postmortem results with results from the stem cell models suggests that these changes in neuronal properties start at early stages of development and may persist throughout development. Additionally, these findings could link the cellular and network phenotype seen in these cultures to the excitation-inhibition (E/I) imbalance which has been proposed as an organizing framework to understand network activity in ASD [168].

Collectively, these studies demonstrate the potential utility of using stem cell models to study ASD by capturing the changes in early brain developmental in ASD at cellular and molecular resolution, but reproducibility and variability remain challenges that each study needs to address. One important caveat is that as many of the individuals used in these studies have complex behavioral phenotypes and comorbidities (i.e., intellectual disability, macrocephaly, epilepsy etc.) and more work will be required to tease apart which of these phenotypes are directly related to the core symptoms of ASD and which may be related to other comorbidities.

## Experimental design and power considerations when using stem cell models to study ASD

While in vitro systems allow us to directly model human brain development, they are only as good as their ability to reliably reproduce processes and cell types occurring in vivo. The first step is, therefore, to create culturing systems that are both scalable and reproducible [83, 84, 86, 169]. A recent study has taken a step in this direction by demonstrating that both scalability and reproducibility can be increased by using a xeno-free approach that simplifies the differentiation protocol by not re-plating cells or embedding them into extracellular matrices [86].

Next, as these in vitro models only approximate in vivo brain development, it is important to ascertain the maturity of the culture used in each study. One system to assess the maturity of the culture uses three different bioinformatic tools to compare the in vitro cultures to in vivo brain development based on their transcriptome [41]. These genome-wide measurements are an important unbiased complement to physiological and morphological analysis of maturation-related phenotypes.

Cell type composition can also have a profound effect on the results. For example, Russo et al. [116] found that the presence of astrocytes derived from iPSCs of individuals with ASD interacted with neurons derived from the same individual to decrease the number of excitatory synapses [116]. It is therefore important to fully characterize the cell types and proportions present in the culturing system either directly by using single-cell technologies such as single-cell RNA sequencing or flow cytometry or indirectly using immunodetection of cell markers. Another aspect highlighted by this study is the importance of having as complete a representation of cell types found in vivo as possible, as this can have a profound change on phenotype [116, 170, 171].

One more related aspect that needs to be considered is that of the brain region being modeled. Many brain regions are involved in different aspects of ASD [7, 172], each with its unique cellular composition and cytoarchitecture. It will therefore be essential to study the specific molecular and cellular changes in ASD in the different brain regions. As an example, one study derived both cortical and olfactory placodal neurons from the same individuals with *SHANK3* mutations [128]. The olfactory placodal neurons had more branched neurite and smaller somas, whereas cortical neurons had shorter neurites [128]. To date, protocols exist for generating many brain regions including the cerebral cortex [74, 83, 169], ventral forebrain [85, 87, 88, 169], cerebellum [173], and midbrain [174]. There are also many protocols to generate specific cell types in 2D, including cortical projection [70], GABAergic neurons [72, 175], and hypothalamic neurons [176]. Combining the different protocols makes it possible to study the interaction between different brain regions [77, 177] and cell types [178, 179] and how these change in ASD. However, it is important to note the tension between the complexity of the system used and the system’s throughput and reproducibility. Each factor and step used in a culture system comes with some intrinsic variability—meaning that the more factors and steps needed, the more variable the system becomes which can negatively impact reproducibility and throughput. This must be taken into account when designing experiments and will depend on the research questions.

**Fig. 1:**
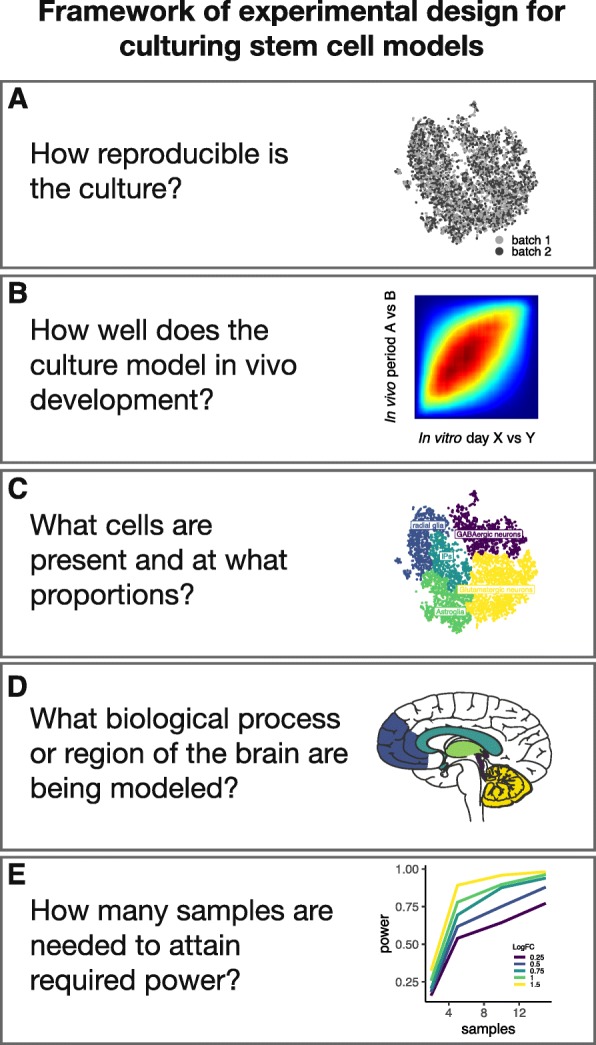
A framework of experimental design and power considerations for culturing stem cell models. **a** Reproducibility can be determined by cell counts, immunocytochemistry, and more recently, single-cell and bulk sequencing. **b** Accuracy of the model can be determined by immunocytochemistry, by single-cell sequencing, and by using tools such as Transition Mapping [41]. **c** Cell proportion can be determined by single-cell sequencing, immunocytochemistry, and flow cytometry. **d** Biological process and region of the brain being modeled can be determined by identifying cell populations using single-cell sequencing and immunocytochemistry as well as by using Transition Mapping [41]. **e** Power can be determined using dedicated tools such as *iPSCpoweR* [180]. Image of brain adapted from Servier Medical art by Servier under Creative Commons License 3.0 (smart.servier.com)

Going beyond the ability of the cultures to reliably model brain development, it is also essential to ensure that the study is suitably powered. Studies to date have not provided a clear power analysis and the number of individuals tend to be relatively low, with most studies having 1–4 affected individuals with rare mutations and 3–8 individuals for studies of individuals with idiopathic forms of ASD (Table 1). A study exploring different experimental designs of disease modeling using iPSC suggests using at least 4 individuals with a known genetic lesion per group, with more individuals increasing the sensitivity of the study design [180]. The authors helpfully developed a framework (with an accompanying software package—*iPSCpoweR*) to assess the number of individuals needed per study [180]. These experimental design and power considerations are summarized in Fig. 1.

## Limitations and future directions of stem cell models for studying ASD

Despite their strengths mentioned above and in Table 2, one has to recognize the limitations of these in vitro models, as is the case with any model system. One clear and obvious conceptual limitation is the lack of the ability to assess behavior. Another, more technical, limitation is the difficulty to collect and maintain large cohorts of iPSC lines, as is evident by the published studies’ fairly small sample sizes (Table 1). This limitation makes it difficult to study the effects of common variation in ASD and limits the utility of these models for non-personalized drug and genetic screening. There are many efforts in the field to overcome this limitation by generating repositories of iPSC lines that will be available to researchers [145, 181–183]. These large repositories will allow researchers to use larger sample sizes to study the effects of genetic background on ASD and will allow them to stratify their studies based on both symptoms and genetic background. Efforts are also being made to increase the throughput of these models to reduce variability and make them more amenable for drug and genetic screens [84, 86, 184].

**Table 2:** Overview of the advantaged and disadvantages of the different in vitro models

**Reprogramed cells**					
	**Epigenetic markers**	**Can be used to study the effect of genetic background**	**Genetic stability**	**Can be used with Crispr**	**Can be cultured into 3D organoids**
**Primary neural precursor cells (pNPCs)**	Unknown	Yes (if genotyped)	High [40]	Yes	No
**Induced neurons (iN)**	Dependent on donor’s age (does not reset)	Yes	High	Yes	No
**Induced pluripotent stem cells (iPSC)**	Mostly embryonic (resets during reprograming)	Yes	Low	Yes	Yes
**Embryonic stem cells (ES)**	Embryonic	Yes (if genotyped)	High	Yes	Yes
**Culture type**					
	**Cellular heterogeneity**	**Reproducibility**	**Can combine different cultures/regions**	**Can be used to study interregional connectivity**	**Level of in vivo brain development modeling**
**2D cultures**	Region-specific cell types/can be enriched for a single cell type	Moderate–high	Yes	No	Low–moderate
**3D cultures—more directed**	Region-specific cell types	Moderate–high	Yes	Yes (when combining different protocols)	Moderate–high (for a specific region)
**3D cultures—less directed**	Non-region-specific cell types	Very low	Unknown	Yes, but likely hindered by variability	Unclear

An additional limitation is that these models diverge from in vivo brain development in a number of aspects. Studies have shown that while human dorsal brain organoids contain cell types and histological structures that reflect in vivo cortex, they differ in their cell proportions and in the complexity of their structural organization [74, 76, 83]. Additionally, these brain organoids can show increased metabolic stress and reduced cell subtype specification [78, 83]. That being said, these issues are surmountable, and further development of these models will need to account for these issues to bring the in vitro models closer to in vivo development. To evaluate the differences between in vitro models and in vivo brain development, single-cell and bulk transcriptomics can provide a quantitative roadmap for unbiased, sensitive comparisons between in vitro and in vivo development [41, 78, 83, 86]. To improve the validity of these stem cell models, new protocols are being developed to generate organoids which include a more complete representation of the cell diversity found in vivo. Such methods include fusing dorsal and ventral forebrain organoids into so called assembloids, to incorporate inhibitory neurons [85, 87, 88], adding growth factors and small molecules to organoid cultures to promote the genesis of oligodendrocytes [185, 186] and adding cells (e.g., microglia) grown separately in 2D [187–189]. Scaffolds are also being developed to increase the structural accuracy of these models [190], a direction which has shown success in modeling other tissues [191, 192].

Another limitation, especially for 3D cultures, is the extended period of time it takes to generate these cultures [77, 81]. For example, one study has shown that to achieve later stages of maturation, including astrocyte maturation, 3D cultures had to be maintained for 9 months [77]. This challenges the feasibility of using these 3D cultures on a very large scale and considerably slows down experimental turnover. One alternative is to use 2D differentiations for these assays, as they have a faster maturation rate [42, 43, 70, 71]. However, as mentioned earlier, these methods diverge significantly from in vivo brain development. Research is, therefore, needed to explore the possibility of accelerating the maturation of the 3D models [193]. One possible way of addressing this limitation is by increasing the oxygen accessibility of the models. A recent study showed that increasing oxygen accessibility to organoids increases their maturation and structural complexity [177]. However, this method is labor intensive and is not representative of the processes in vivo. A more physiologically relevant method would be to incorporate vasculature and a functional blood brain barrier [194] which would allow for oxygen and nutrients to permeate the entire organoid. An analogous method is to transplant the organoids into a host organism such as mice or rats. This method, while having a low throughput, allowed the organoids to progressively mature and form intact networks between the organoid and the host [78, 195, 196].

## Conclusion

The promise of stem cell models to study both typical and non-typical human brain development is already coming to fruition. However, careful consideration is needed when designing experiments using these models by taking into account both biological, (i.e., maturity and cell composition) and technical considerations (number of samples, protocol variability, differentiation time) for these models to meet their full potential.

## Data Availability

Not applicable

## Abbreviations

ASD: Autism spectrum disorder
GWAS: Genome wide association study
eQTL: Expression quantitative trait loci
CMV: Cytomegalovirus
NDD: Neurodevelopmental disorders
phNPC: Primary human neural precursor cells
iN: Induced neuron
ESC: Embryonic stem cell
iPSC: Induced pluripotent stem cell
CNV: Copy number variant
SNV: Single nucleotide variant
2D: 2 dimensional
3D: 3 dimensional
DEG: Differential expressed genes
EPSC: Excitatory postsynaptic current
sEPSC: Spontaneous excitatory postsynaptic current
mEPSC: Miniature excitatory postsynaptic current
NPC: Neuron precursor cell
